# 824 2-Ocytl Cyanoacrylate – a Split Thickness Skin Graft Donor Site Dressing?

**DOI:** 10.1093/jbcr/iraf019.355

**Published:** 2025-04-01

**Authors:** Nathan Tanoue, Jason Heard, Kathleen Romanowski, Tina Palmieri, Soman Sen

**Affiliations:** Firefighters Burn Institute Regional Burn Center - University of California Davis; University of California Davis; University of California Davis and Shriners Hospitals for Children; University of California Davis and Shriners Hospitals for Children; University of California Davis and Shriners Hospitals for Children

## Abstract

**Introduction:**

2-octyl cyanoacrylate tissue adhesive is commonly in use to close surgical wounds. It is reported to have antibacterial, hemostatic, and tissue sealant properties and has been utilized in a wide variety of off-label applications. In autologous skin grafting, it has been described as a method to secure meshed split thickness skin grafts (STSG). To our knowledge it has never been described as a dressing for the donor site in the literature. Here we present five patients with 2-octyl cyanoacrylate resin applied to the STSG donor site as a novel dressing.

**Methods:**

This is a case series of five patients who underwent split-thickness skin grafting with 2-octyl cyanoacrylate application to the donor site as a novel dressing at a single academic hospital.

Patients were male between the ages of 17 and 78 with < 5% TBSA partial thickness burns requiring STSG from a single thigh donor site.

Pictures of the donor sites taken on daily rounds and dressing changes were evaluated as well as pain scores and total opioid requirement.

**Results:**

All patients reported “mild” or “moderate” pain at the donor sites through the first postoperative week. 4 of 5 patients also had fascio-iliac nerve block catheter inserted prior to the start of operation.

There was no report of infection at the donor sites.

One patient’s tissue adhesive glue accumulated some serous fluid and began to come off at day 6 and was removed. The underlying tissue was moist, healthy and in an expected state of healing.

There was no bleeding requiring intervention at the donor sites.

**Conclusions:**

2-Octyl cyanoacrylate as a donor site dressing for split thickness skin grafts remains an intriguing option that deserves further research. Our case reports suggest this dressing may improve postoperative pain, reduces dressing change requirements, and maintain a moist environment for healing.

**Applicability of Research to Practice:**

2-Octyl cyanoacrylate dressing of STSG donor sites represents an option that provides antibacterial and hemostatic properties while minimizing the labor and cost of repeated dressing changes.

**Funding for the Study:**

N/A

